# A Multilabel Text Classifier of Cancer Literature at the Publication Level: Methods Study of Medical Text Classification

**DOI:** 10.2196/44892

**Published:** 2023-10-05

**Authors:** Ying Zhang, Xiaoying Li, Yi Liu, Aihua Li, Xuemei Yang, Xiaoli Tang

**Affiliations:** 1 Institute of Medical Information Chinese Academy of Medical Sciences Beijing China

**Keywords:** text classification, publication-level classifier, cancer literature, deep learning

## Abstract

**Background:**

Given the threat posed by cancer to human health, there is a rapid growth in the volume of data in the cancer field and interdisciplinary and collaborative research is becoming increasingly important for fine-grained classification. The low-resolution classifier of reported studies at the journal level fails to satisfy advanced searching demands, and a single label does not adequately characterize the literature originated from interdisciplinary research results. There is thus a need to establish a multilabel classifier with higher resolution to support literature retrieval for cancer research and reduce the burden of screening papers for clinical relevance.

**Objective:**

The primary objective of this research was to address the low-resolution issue of cancer literature classification due to the ambiguity of the existing journal-level classifier in order to support gaining high-relevance evidence for clinical consideration and all-sided results for literature retrieval.

**Methods:**

We trained a multilabel classifier with scalability for classifying the literature on cancer research directly at the publication level to assign proper content-derived labels based on the “Bidirectional Encoder Representation from Transformers (BERT) + X” model and obtain the best option for X. First, a corpus of 70,599 cancer publications retrieved from the Dimensions database was divided into a training and a testing set in a ratio of 7:3. Second, using the classification terminology of International Cancer Research Partnership cancer types, we compared the performance of classifiers developed using BERT and 5 classical deep learning models, such as the text recurrent neural network (TextRNN) and FastText, followed by metrics analysis.

**Results:**

After comparing various combined deep learning models, we obtained a classifier based on the optimal combination “BERT + TextRNN,” with a precision of 93.09%, a recall of 87.75%, and an *F*_1_-score of 90.34%. Moreover, we quantified the distinctive characteristics in the text structure and multilabel distribution in order to generalize the model to other fields with similar characteristics.

**Conclusions:**

The “BERT + TextRNN” model was trained for high-resolution classification of cancer literature at the publication level to support accurate retrieval and academic statistics. The model automatically assigns 1 or more labels to each cancer paper, as required. Quantitative comparison verified that the “BERT + TextRNN” model is the best fit for multilabel classification of cancer literature compared to other models. More data from diverse fields will be collected to testify the scalability and extensibility of the proposed model in the future.

## Introduction

### Background

According to the World Health Organization (WHO) reports, cancer is one of the leading causes of death worldwide, accounting for nearly 10 million deaths in 2020 [1]. With cancer emerging as the greatest threat to human life, there is a rapid growth in the volume of literature published in the cancer field. The trend of disciplinary convergence has led to publications requiring labels from multiple subjects. Consequently, there is increasingly more demand for accurate cancer literature classification for retrieval, evidence support, academic analysis, and statistical evaluation in order to support clinical research, precision medicine, and discovery of interdisciplinary cancer research [2,3] by forecasting trends and hotspot statistics.

Text classification is the process of assigning specific labels to the literature based on individual features. The current methods of classifying the literature can be divided into 3 groups: mapping based, subject information based, and machine learning based [4-6]. Recently, an increasing number of studies have experimented with deep learning to enhance the effects of text classification [4]. Most of the existing literature classification (eg, Web of Science, Scopus [7-11]) is usually carried out at the journal level, that is, all papers in a given journal get the same labeling categories as the hosted journal. However, given that interdisciplinary research is increasing in the cancer field [12], there is a need for a more precise classifier, as papers from a journal always present a diverse range of topics [13,14]. Moreover, literature classification at the journal level can no longer adapt to the dynamics of newly developing subjects and fully characterize text features.

### Related Works

#### Development of Text Classification Technology

Text classification technology has undergone rapid development from expert systems to machine learning to finally deep learning [15]. Maron [16] published the first paper on automatic text classification in 1961. By the end of the 20th century, machine learning had matured into a fully developed field [17]. Joachims [18] established the bag-of-words model to transform text into a vector with a fixed length and then selected features using the information gain criterion to achieve dimensionality reduction, eventually training the feature vector iteratively using a support vector machine (SVM) classifier. Rasjid [19] focused on data classification using k-nearest neighbors (k-NN) and naive Bayes. Liang et al [20] improved the feature recognition of the literature using appropriate clusters and the introduction of differential latent semantics index (DLSI) spaces. In 2006, with the rapid development of deep learning, text classification research based on deep learning gradually replaced traditional machine learning methods and became the mainstream, with wide applications in numerous tasks [4].

The deep learning–based text classification method adopts word vectors (eg, GloVe [21] and word2vec [22]) for word semantic representation [23], and subsequently, various deep neural network (DNN)–based text classification methods develop. Convolutional neural networks (CNNs) [24] were originally constructed for image processing and have been broadly used for text classification [25-27]. Due to the computational complexity, deep network gradient disappearance, and short text content of a CNN, a series of optimized models were gradually derived to address these issues, including FastText [28], deep pyramid convolutional neural networks (DPCNNs) [29], knowledge pyramid convolutional neural networks (KPCNNs) [30], and text convolutional neural networks (TextCNNs) [26]. Especially, the TextCNN is a simple, shallow network and requires only a small number of hyperparameters for fine-tuning. Compared with CNNs, recurrent neural networks (RNNs) can easily implement multilayer superposition to construct a multilayer neural network [31], such as multilayer long short-term memory (LSTM) [32] or multilayer gate recurrent unit (GRU). In terms of improvements to RNNs, the text recurrent neural network (TextRNN) uses a multitask learning framework to jointly learn across multiple related tasks; a deep recurrent neural network (DRNN) [23] incorporates position invariance into an RNN, captures local features to find the optimal window size, and then achieves marked improvements over RNN and CNN models. All these models have laid the foundation for follow-up studies.

Bidirectional Encoder Representation from Transformers (BERT) has emerged as a new linguistic representation model by the introduction of attentional mechanisms, which have been broadly applied in machine translation [32], image description generation [33], machine reading comprehension [34], and text classification [35]. Being a bidirectional encoder model based on a transformer, BERT became an important advancement of natural language processing, especially text classification. For example, Shen et al [36] attempted to train a Chinese corpus–based BERT_base_ model for the classification of the literature on Chinese social science and technology and also explored its application to practical production. In addition, Lu and Ni [37] developed a multilayer model for patent classification using the combination “BERT + CNN,” while Liu et al [38] proposed a sentence-BERT for hierarchical clustering of literature abstracts. However, the accuracy of applying universal language models directly to the biomedical field is not sufficient, and this motivated studies to train the biomedical BERT from scratch. Typical instances are BioBERT pretrained on PubMed citations and PubMed Central (PMC) full text [39] and PubMedBERT obtained with mixed-domain pretraining using PubMed text and clinical notes [40]. Up to now, BioBERT and PubMedBERT have achieved success in named entity recognition, extraction of relationships between entities, entity normalization [41,42], *International Classification of Diseases* (ICD) autocoding [43], and its multilabel classification (MLC) [44]. These studies afford us lessons that merit attention.

#### Multilabel Text Classification

The deep learning model has contributed to the success of MLC due to its dynamic representation learning and end-to-end learning framework. Multilabel text classification (MLTC) is the application of MLC to the task of text classification and the assigning of a set of targeted labels to each sample [45], which has been part of the longstanding challenge in both academia and industry. In the biomedical domain, Du et al [46] proposed an end-to-end deep learning model ML-Net for biomedical text, while Glinka et al [47] focused on a mixture of feature selection methods, filter, and wrapper methods. In addition, Hughes et al [48] tried to classify medical text fragments at the sentence level based on a CNN, and Yogarajan et al [49] used a multilabel variant of medical text classification to enhance the prediction of concurrent medical codes. Automatic question-and-answer systems and auxiliary decision-making are the 2 typical applications of MLTC. For example, Wasim et al [50] proposed a classification model for multilabel problems in the flow of biomedical question-and-answer systems with factlike and listlike questions. Similarly, Baumel et al [51] presented a hierarchical attentional bidirectional gated cyclic unit that used attention weights to better understand the sentence and word with the greatest impact on the decision.

In this study, we adopted MLTC technology to classify cancer publications for better retrieval, academic analysis, and statistical evaluation. We introduced a method of multilabel classification for cancer research at the publication level based on the “BERT + X” model. BERT is a learning model that can migrate to other tasks to obtain better outcomes, which was pretrained on a large and easily accessible data set, and X is a deep learning model that can capture semantic features of text accurately. The combined model was trained on a corpus from a multilabel publication database, and then cancer publications were classified into appropriate categories directly at the publication level instead of the journal level.

## Methods

### Study Design

**Figure 1:**
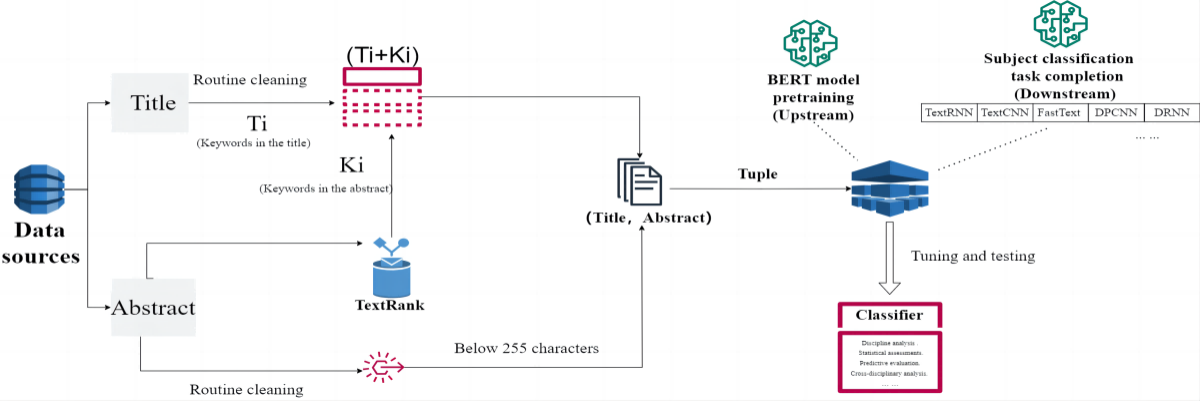
Study framework. BERT: Bidirectional Encoder Representation from Transformers; DPCNN: deep pyramid convolutional neural network; DRNN: deep recurrent neural network; TextRNN: text recurrent neural network; TextCNN: text convolutional neural network.

This study mainly aimed to train a deep learning–based multilabel classifier for cancer literature classification at the publication level. The overall framework of the study is illustrated in Figure 1. First, a corpus of titles and abstracts of cancer publications retrieved from the Dimensions database were divided into a training and a testing set in a ratio of 7:3 after preprocessing. Second, to capture sufficient text features for multilabel classification, the titles and abstracts were taken separately as 2 independent layers, which were called the “tuple” in this study. Finally, “BERT + X” classifiers based on 5 deep learning models were trained; X refers to “TextRNN,” “TextCNN,” “FastText,” “DPCNN,” and “DRNN.” The performance of the candidate classifiers was compared quantitatively in terms of 3 conventional metrics in order to identify the optimal model for classifying cancer literature.

### Data Collection and Preprocessing

Refined data with semantic and context features are the basis of deep learning model training. In this study, to train a lightweight, compatible, and high-applicability classifier for text classification, we first preprocessed cancer literature data to extract features and sequential semantic information. The classification terminology maintained by the International Cancer Research Partnership (ICRP), called cancer type (CT), was used as standard labels to characterize individual cancer studies in terms of 62 CTs. The ICRP CT has been linked to the ICD maintained by WHO [52] and is increasingly gaining rapid recognition worldwide. Moreover, the ICRP CT has been applied to several international databases for labeling cancer literature and research documents with fine granularity. A typical example is the Dimensions database (Digital Science & Research Solutions, Inc), which covers more than 135 million publications, 6.5 million grants, and 153 million patents, providing a collaborative path to enhanced scientific discovery with transparent data sources. Importantly, cancer publications with ICRP CT–classified labels from the Dimensions database provide a way to prepare annotation data for model training.

#### Construction of the Corpus and Balanced Sampling

**Figure 2:**
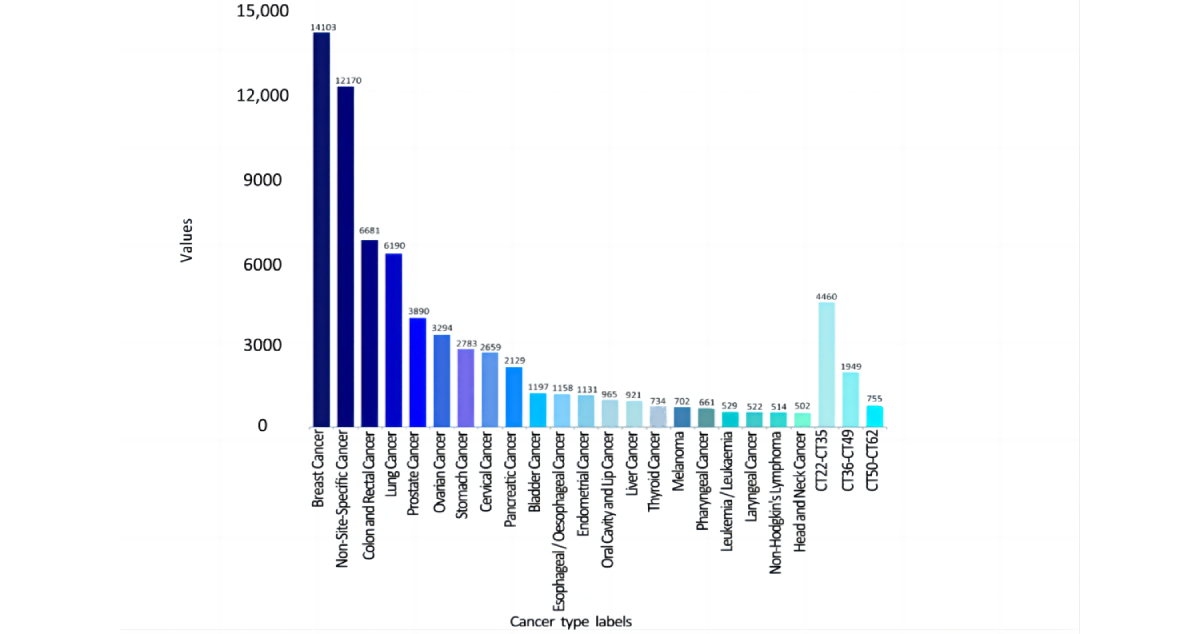
Original sample distribution of CTs. CT: cancer type.

A set of 70,599 publications from 2003 to 2022 was randomly sampled from the Dimensions database using the keyword “cancer,” along with the ICPR CT labels for each publication. Figure 2 shows the distribution of different CTs among the corpus data. Here, to intuitively demonstrate the volume distribution in the 62 different cancer categories, we ranked the categories in descending order of the number of corresponding publications included. Categories with sample sizes larger than 500 were listed separately, while the remaining 41 (66.13%) categories were grouped into 3 classes: CT22-CT35, n=14 (34.15%) categories; CT36-CT49, n=14 (34.15%); and CT50-CT62, n=13 (31.71%). The top 9 (14.52%) categories (breast cancer, non-site-specific cancer, colon and rectal cancer, lung cancer, prostate cancer, ovarian cancer, stomach cancer, cervical cancer, and pancreatic cancer) accounted for 76.35% (53,899/70,599) of the total corpus. Obviously, the distribution of labeling data was uneven, and the top 9 labels contained more than three-fourths of the total corpus. Once the original corpus was directly used to train the deep learning model without balanced sampling, the resulting classifier would cause overfitting due to a few categories with excessive volume and fail to be generalized in practical use.

To avoid the degradation of precision caused by overfitting, we balanced the data sampling before model training. First, we ranked the cancer categories in descending order of the number of corresponding publications. Next, a threshold (70 in our study) used for setting the sampling index was obtained after multiple testing, followed by calculation of the index using categories with more than 70 publications. The resulting mean , median, variance, standard deviation of the number of samples contained in each category were recorded as follows: mean=1127, median=282, variance=5,889,927, and standard deviation=2427. Here, the median was selected as the initial index according to the actual distribution of sampling data. Considering that 30% of the corpus was used as the testing set, the final index was set to 500 in terms of the number of publications for a competent training set. Therefore, categories whose number of publications exceeded the final index were balanced and down-sampled separately, which means that 500 publications from each category were randomly extracted for a uniform corpus.

Table 1 shows part of the balanced sampling results. It is clear that the optimized corpus contributed to a reduction in the adverse effects of overfitting and was subsequently used for keyword extraction and model training.

**Table 1:** Example of balanced sampling of top 21 categories (N=70,599 publications).

Sequence number	ICRP CT^a^	ICRP code^b^	ICD-10^c^ code	Original sample^d^, n (%)	Balanced sample^e^, n (%)
1	Breast cancer	7	C50	14,103 (19.98)	500 (3.55)
2	Non-site-specific cancer	2	N/A^f^	12,170 (17.24)	500 (4.11)
3	Colon and rectal cancer	64	C18, C19, C20	6681 (9.46)	500 (7.48)
4	Lung cancer	28	C34, C45	6190 (8.77)	500 (8.08)
5	Prostate cancer	42	C61	3890 (5.51)	500 (12.85)
6	Ovarian cancer	66	C56	3294 (4.67)	500 (15.18)
7	Stomach cancer	51	C16	2783 (3.94)	500 (17.97)
8	Cervical cancer	9	C53	2659 (3.77)	500 (18.80)
9	Pancreatic cancer	37	C25	2129 (3.02)	500 (23.49)
10	Bladder cancer	3	C67	1197 (1.70)	500 (41.77)
11	Esophageal/oesophageal cancer	12	C15	1158 (1.64)	500 (43.18)
12	Endometrial cancer	11	C54	1131 (1.60)	500 (44.21)
13	Oral cavity and lip cancer	36	C00, C01, C02, C03, C04, C05, C06, C09	965 (1.37)	500 (51.81)
14	Liver cancer	23	C22	921 (1.30)	500 (54.29)
15	Thyroid cancer	54	C73	734 (1.04)	500 (68.12)
16	Melanoma	29	C43	702 (0.99)	500 (71.23)
17	Pharyngeal cancer	61	C14.0	661 (0.94)	500 (75.64)
18	Leukemia/leukaemia	27	C91, C92, C93, C94, C95	529 (0.75)	500 (94.52)
19	Laryngeal cancer	26	C32	522 (0.74)	500 (95.79)
20	Non-Hodgkin’s lymphoma	35	C82, C83, C84, C85, C96.3	514 (0.73)	500 (97.28)
21	Head and neck cancer	21	C76.0	502 (0.71)	500 (99.60)

^a^ICRP CT: International Cancer Research Partnership Cancer Type; here, “ICRP CT” denotes the label.

^b^“ICRP code” refers to the label code.

^c^ICD-10: International Classification of Diseases, Tenth Revision; this is the ICD code linked to the appropriate ICPR CT.

^d^“Original sample” represents the number of publications obtained directly from the Dimensions database.

^e^“Balanced sample” means the number of publications after balanced sampling.

^f^N/A: not applicable.

#### Construction of a Tuple Consisting of a Title and an Abstract

The corpus of cancer publications consisted of titles and abstracts in English, while the title and abstract of each publication was saved separately for ease of use. Generally, the title is a short sentence of a confined length to express an independent meaning, which has a high rate of conformity regarding the content. Comparatively, an abstract is also valuable, since it clearly and accurately summarizes the main content of the publication by expressing its purpose, methods, results, and conclusions. In this study, both title and abstract were independently used to train a 2-layer classifier based on their semantic and context features, called a tuple for simplicity.

#### Keyword Extraction From the Abstracts of Cancer Publications

In this study, in contrast to the abstract layer, the operation of the title layer mainly focused on keyword training. However, the number of valid keywords contained in titles is quite limited. To improve feature representation and model performance, more keywords were extracted from the abstracts and merged with the title layer. The TextRank algorithm [53] was adopted for keyword extraction from the abstracts, taking advantage of the co-occurring semantics information between words from the given sentences.

**Figure 3:**
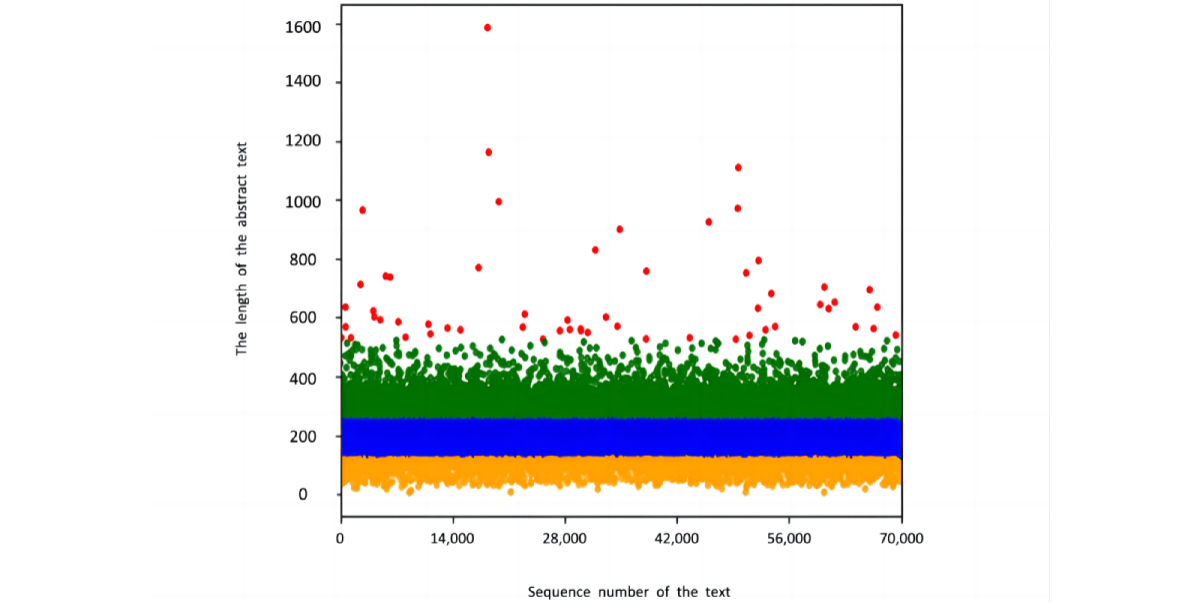
Scatter plot of distribution of abstract length.

A lightweight classifier was desired in this study, so the length of the abstracts needed to be controlled to keep a balance between the running speed, effectiveness, and volume occupied. To ascertain the most appropriate abstract length, we analyzed the scatter plot of the abstract length distribution, as shown in Figure 3. Here, the horizontal axis represents the serial number of publications, the vertical axis represents the length of the publication abstracts, and the red (length>512), green (256<length≤512), blue (128<length≤256), and orange (length≤128) colors represent different abstract lengths separately. Among them, the blue zone was evenly distributed and had a high proportion, the red zone had the least proportion, and the green and orange zones were comparable.

After statistical analysis, the maximum length of the abstracts was set to 256 characters mainly for 3 reasons. First, 256 is the 8th power of 2, which facilitates machine understanding after tuning [53]. Second, we wanted to collect the most valid information as much as possible, while reducing sparsity. Third, we wanted to avoid the learning of shallow networks, which are equivalent to follow-up layers when training (ie, gradient disappearance).

### Training a Model for MLCT

#### Upstream Pretrained Language Models

Pretraining generally refers to putting a large amount of low-cost collected training data together, learning the commonalities of the data, and then tuning the model that has the commonalities with a small amount of labeled data of a specific domain. Therefore, pretrained language models start from the commonalities and learn the special parts of the specific task. BERT is a successful model pretrained on Wikipedia and a book corpus via self-supervision tasks, and fine-tuning benefits downstream tasks. Being a pretrained language model based on the bidirectional transformer encoder architecture, BERT uses sentence-level negative sampling to obtain sentence representation/sentence pair relationships. In addition, BERT takes advantage of the transformer model instead of LSTM for expressive and temporal efficiency, as well as the masked language model to extract contextual features. We used BERT to handle specific natural language processing tasks downstream to produce word vectors in the pretraining stage. During the fine-tuning, we then completed data training for the pretrained BERT through the output layer based on cancer publications in order to save time and improve accuracy.

#### Downstream Classification Model Training

To complete the actual task of downstream natural language processing based on the fine-tuned upstream procedure, we tried to train several preliminary language models and then chose one as the optimal model for our classifier according to its all-round performance. The “BERT + X” pattern was adopted for the classifier to determine the optimum option for X and the best way to combine BERT and X. According to the actual scenario and expert consultations, including the length, tightness of context, and multidisciplinarity, 5 models suitable for cancer publications were compared for the classification model: TextCNN, TextRNN, FastText, DPCNN, and DRNN. Eventually, the definitive combined classifier would come out dependent on the comprehensive performance analysis of these 5 models.

**Figure 4:**
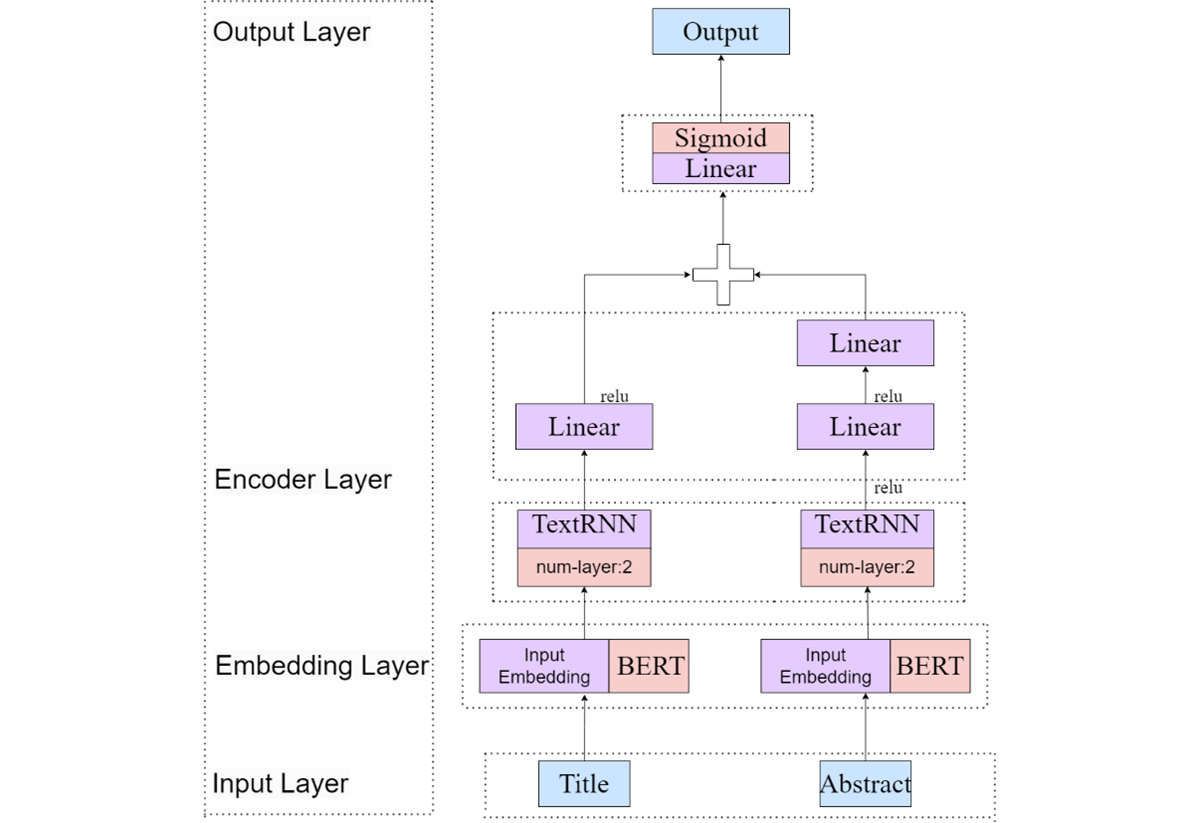
Structure of the classification model. BERT: Bidirectional Encoder Representation from Transformers; TextRNN: text recurrent neural network.

The structure of the classification model is shown in Figure 4, where the TextRNN is selected as a representation of the 5 models, for example. Here, the title and abstract were input into the title layer and the abstract layer, respectively, while the word vectors were converted by BERT and passed to the encoder layer. The word vectors output from the title and abstract layers were stitched together and then transferred to the fully connected layer for normalization, while the final output of multilabel classification was generated by the sigmoid layer with the activation function.

### Testing and Verification

To evaluate the performance of the trained classifier, a subset of the testing sample was selected from the corpus, which covered all 62 classification labels defined in the ICRP CT. We applied 3 frequently used indexes, namely precision, recall, and the *F*_1_-score, to verify the classification results of the 5 models. Here, the *F*_1_-score is the harmonic mean of precision and recall; the larger the *F*_1_-score, the better the performance of the classification model. The quantitative indexes of the 5 “BERT + X” models were compared numerically and independently to choose X.

## Results

### Quantitative Analysis of the Performance of Classification Models

The test results of the combined classification downstream models are shown in Table 2, where we compared performance on 3 aspects (precision, recall, *F*_1_-score) of 5 classification models (BERT + TextRNN, BERT + TextCNN, BERT + FastText, BERT + DPCNN, and BERT + DRNN). All metrics of BERT + TextRNN were consistently at a high level, with a precision of 93.09%, a recall of 87.75%, and an *F*_1_-score of 90.34%. Here, BERT was directly used for fine-tuning training, combined with the TextRNN for multilabel classification. After adjusting and testing the parameters several times, the best parameters were obtained and are shown in Table 3.

**Table 2:** Performance comparison of 5 different “BERT^a^ + X” models.

Model	Precision (%)	Recall (%)	*F*_1_-score (%)
BERT + TextRNN^b^	93.09	87.75	90.34
BERT + TextCNN^c^	84.19	79.69	81.88
BERT + FastText	93.05	75.73	83.50
BERT + DPCNN^d^	81.78	75.00	78.25
BERT + DRNN^e^	88.98	53.05	66.47

^a^BERT: Bidirectional Encoder Representation from Transformers.

^b^TextRNN: text recurrent neural network.

^c^TextCNN: text convolutional neural network.

^d^DPCNN: deep pyramid convolutional neural network.

^e^DRNN: deep recurrent neural network.

**Table 3:** Parameters of the optimal “BERT^a^ + TextRNN^b^” model.

Parameter	Value
Num_ train_ epochs	200.0
Max_ seq_ length	256
leaning_ rate	0.0001
train_ batch_ size	32
Predict_ batch_ size	32
Drop	0.5
Dense1	256
Dense2	62
TextRNN	256×2
LTSM^c^_UNITS	5
BERT_OUTDIM	768

^a^BERT: Bidirectional Encoder Representation from Transformers.

^b^TextRNN: text recurrent neural network.

^c^LSTM: long short-term memory.

### Supplementary Analysis of the Model Structure

The proposed classification model takes the title and abstract of a publication as independent input, namely a tuple, as mentioned in the “Methods” section. To verify the effectiveness of the tuple input of the trained model, we conducted a set of comparison experiments based on “BERT + TextRNN.” Especially, “2 tuples and 2 levels” represents the model that took the title and abstract as 2 levels of the training model separately, “1 unit and 1 level” represents the model that combined the title and abstract as whole-text input for training, and “the title alone” and “the abstract alone” represent the models that took the title or the abstract alone as input, respectively. Table 4 records the performance of different models from supplementary experiments, where the “2 tuples and 2 levels” model was superior, with a precision of 93.09%, a recall of 87.75%, and an *F*_1_-score of 90.34%. The reason is that when applying the title or abstract alone to train the classification model, feature reduction occurs, which further leads to inferior performance of classification. In addition, compared with the “1 unit and 1 level” model taking the title and abstract as a 1-part text input, the “tuple and 2 levels” model enhanced the specificity of feature extraction. Notice that the title and abstract make different contributions to the subject of publication, and the classification model will lose the sufficient feature of the abstract if it is not trained separately. Eventually, the classification of the tuple input was elected for the proposed classification model. This is also identical to most of the subject-based literature processing, which take the title and abstract as independent text, such as subject indexing.

**Figure 5:**
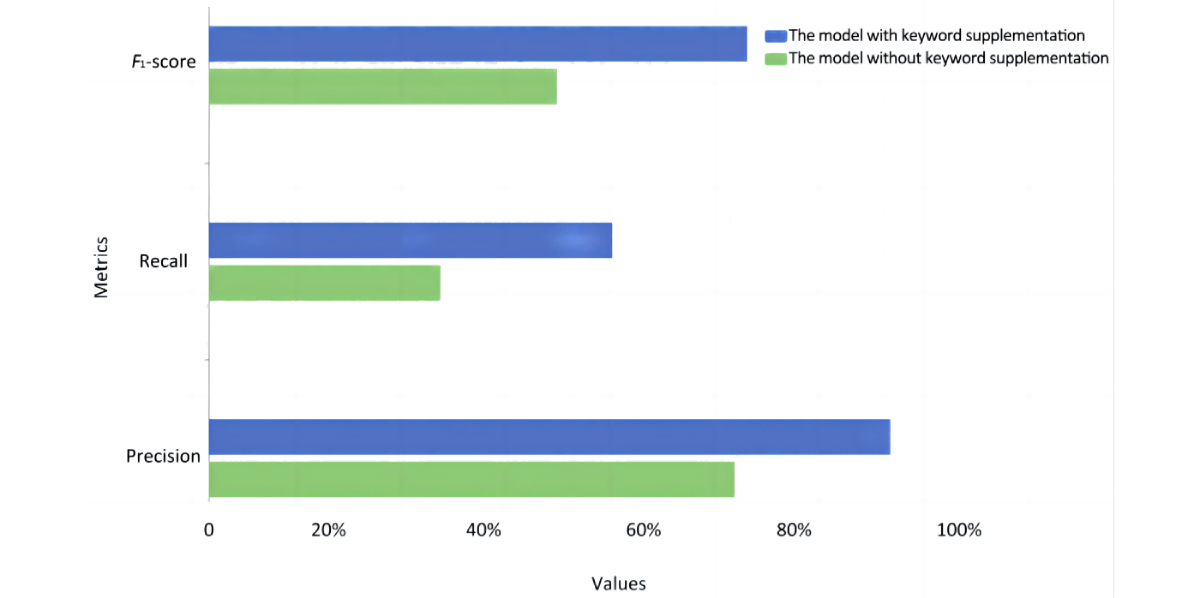
Comparison of the model with and without keyword supplementation.

In addition, the proposed classification model used the TextRank algorithm to extract keywords from the abstract and supplement the title layer with them. To demonstrate the necessity and effectiveness of this step, Figure 5 shows the numerical indexes of the classification model with and without keyword extraction and supplementation. Here, the performance of the model with TextRank keyword extraction is shown in blue, and the results with direct training using titles and abstracts separately are plotted in green. It is obvious that the model without keyword supplementation had a lower performance in recall, precision, and the *F*_1_-score, which confirms the efficiency and effectiveness of the proposed classification model from a different perspective.

**Table 4:** Comparison of different types of input.

Input	Precision (%)	Recall (%)	*F*_1_-score (%)
2 tuples and 2 levels	93.09	87.75	90.34
1 unit and 1 level	82.32	79.78	81.04
The title alone	44.37	31.54	36.88
The abstract alone	85.50	79.79	82.55

### Comprehensive Analysis of the Multilabel Classification

**Figure 6:**
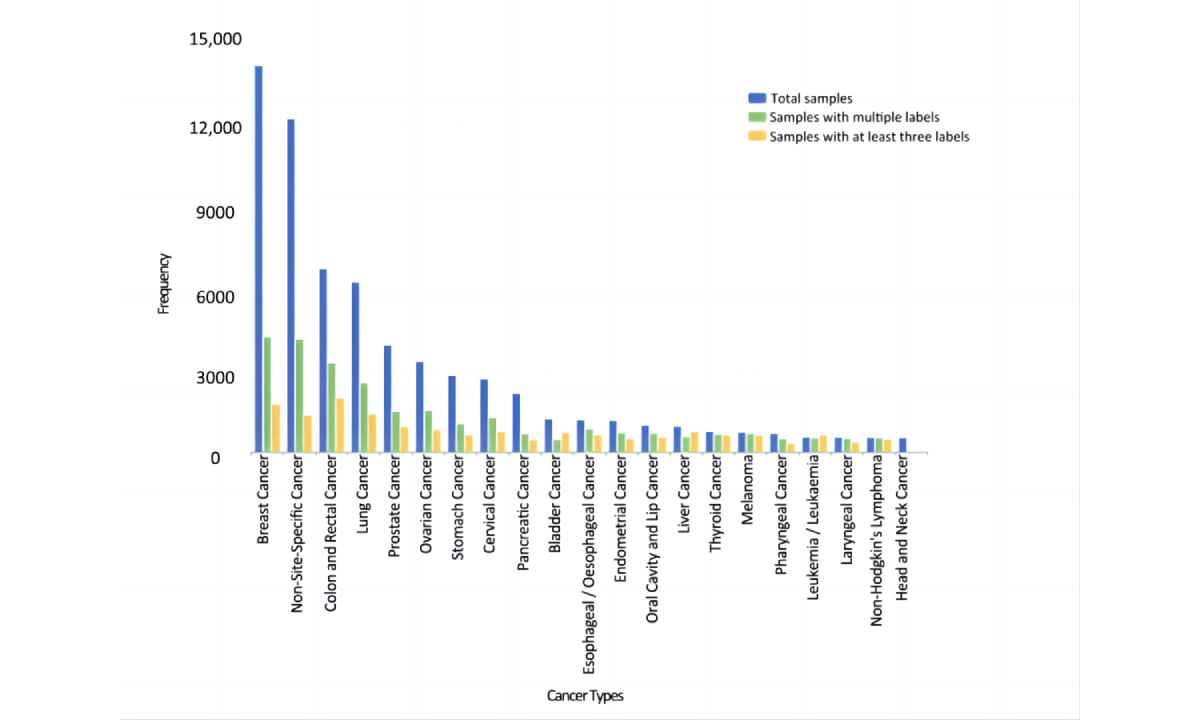
Comparison of samples with multilabels.

To explore whether there was a particular regularity in the distribution of multiple labels among different categories, the proportion of multilabel publications was statistically analyzed for classifier training. In total, 15,296 (21.67%) of the 70,599 publications had 2 or more labels, that is, more than one-fifth of the publications were multilabel ones. In addition, the categories were counted based on the characteristics of the number of labels (Figure 6) to visualize multilabel distributions. Here, we listed the top 20 categories by the volume of the publications included. The blue color refers to the total number of samples collected under a specific category, the green color is the number of samples with multiple labels under that category, and the yellow color denotes those with at least 3 labels. The categories with fewer samples had a higher ratio of multiple labels, and multiple labels had different characteristics among different categories. Analysis for a deeper relationship between multiple labels is necessary.

There are principally 2 roles of comprehensive analysis of multilabel publications. First, it highlighted the process of balanced sampling. Since part of the publications belonged to multiple categories and some of the categories had a high co-occurrence frequency compared to other categories, direct model training on the original corpus would lead to overfitting due to uneven distribution of samples. This is why we selected multilabel papers instead of those with a single label in order to obtain a balanced sample for classifier training. Second, the multilabel publications revealed the potential semantic correlation of texts, which provided a direction for the analysis of data characteristics. Based on the co-occurrence correlation and distribution between different categories, the semantic features were further characterized and the proposed classification model extended to other data with the same characteristics.

To explore the inherent correlation between multiple labels, we selected 2500 multilabel publications from the corpus for characteristics analysis. Specifically, samples with 2 labels accounted for 59.04% (1476/2500) and samples with at least 3 labels accounted for 40.96% (1024/2500) of all publications. Table 5 lists part of the analysis results. For instance, different CTs often co-occurred for statistical surveys in the literature review with a weak association.

The correlation strength of multiple labels of cancer publications were independently reviewed and assessed by 3 biocurators with relevant knowledge. Concretely, a publication with 2 labels and a clear semantic correlation within the corresponding subject classification labels was interpreted as “1,” while a publication with 3 or more labels and more than two-third of the labels holding an obvious semantic association were also considered as “1”. Once the 2 biocurators reached the same results, that specific publication was passed into the “review completed” data set. When they had different opinions, the corresponding publication was annotated as “pending review.” After the first round of reviewing, the “pending review” data set was discussed together for the second time, and a third biocurator was invited for confirmation and agreement.

**Figure 7:**
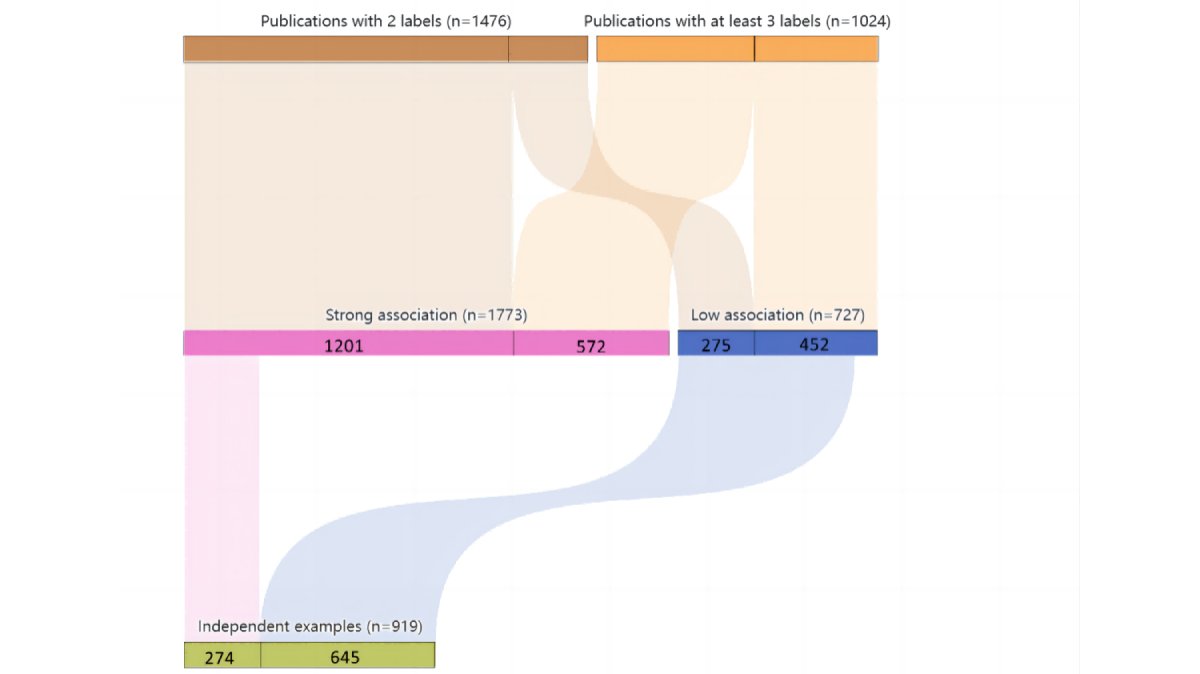
Relational mapping of multilabel publication distributions.

Figure 7 shows specific numbers of labeled publications with interlabel correlation, where the “strong association” zone consists of publications whose co-occurrence labels had explicit links between semantics, the “low association” zone consists of publications whose co-occurrence labels did not have clearly semantic links, and the “independent examples” zone consists of publications whose cancer labels were taken as single entities or independent examples for observation without intrinsic correlations. Of the 1476 publications with 2 labels, 1201 (81.37%) had a strong association, while 572/1024 (55.86%) publications with at least 3 labels had a low association. Among the publications with lowly correlated labels, 718/1024 (70.12%) took the different categories of cancers as a single entity or independent examples for observation without intrinsic correlations. We noticed some possible influence on the association distributions for training. In addition, the relationship between 2 labels in a publication was stronger than that among 3 or more labels, which justifies the demand to classify publications by subject at the publication level. Therefore, the strength of interlabel association could achieve the effect of assisting decision-making after multilabel classification to further support clinical diagnosis and treatment. In the future, we will carry out knowledge mining based on the existing interlabel semantic network and strengthen the training of interlabel association to improve the performance of the proposed classification model.

**Table 5:** Examples of data evaluated by experts.

Sequence number	Title	Labels, n	Correlation strength^a^
1	Temporal Trends of Subsequent Breast Cancer Among Women With Ovarian Cancer: A Population-Based Study [54]	2	1
2	Clinical Characteristics and Survival Outcomes of Patients With Both Primary Breast Cancer and Primary Ovarian Cancer [55]	2	1
3	Secondary Malignancies in Long-Term Ovarian Cancer Survivors: Results of the “Carolin Meets HANNA” Study [56]	2	1
4	Trends in Participation Rates of the National Cancer Screening Program among Cancer Survivors in Korea [57]	3	0
5	Increasing Trends in the Prevalence of Prior Cancer in Newly Diagnosed Lung, Stomach, Colorectal, Breast, Cervical, and Corpus Uterine Cancer Patients: A Population-Based Study [58]	4	1
6	Cancer Registration in China and Its Role in Cancer Prevention and Control [59]	3	0
7	Cancer Incidence, Mortality, and Burden in China: A Time‐Trend Analysis and Comparison With the United States and United Kingdom Based on the Global Epidemiological Data Released in 2020 [60]	5	0
8	Excess Costs and Economic Burden of Obesity-Related Cancers in the United States [61]	3	1
9	Cancer Attributable to Human Papillomavirus Infection in China: Burden and Trends [62]	4	0
10	Excess Costs and Economic Burden of Obesity-Related Cancers in the United States [63]	3	1
11	Cancer Awareness in the General Population varies With Sex, Age and Media Coverage: A Population-Based Survey With Focus on Gynecologic Cancers [64]	5	0
12	Public Attitudes Towards Cancer Survivors Among Korean Adults [65]	3	0
13	Importance of Hospital Cancer Registries in Africa [66]	2	0
14	Correlation Between Family History and Characteristics of Breast Cancer [67]	2	1
15	Familial Aggregation of Early‐Onset Cancers [68]	3	0
16	Trends in Regional Cancer Mortality in Taiwan 1992–2014 [69]	6	0
17	Statin Use and Incidence and Mortality of Breast and Gynecology Cancer: A Cohort Study Using the National Health Insurance Claims database [70]	4	1
18	Management of Breast Cancer Risk in BRCA1/2 Mutation Carriers Who Are Unaffected With Cancer [71]	2	1
19	Association Between Diabetes, Obesity, Aging, and Cancer: Review of Recent Literature [72]	4	0
20	The European Cancer Burden in 2020: Incidence and Mortality Estimates for 40 Countries and 25 Major Cancers [73]	3	0

^a^In the last column, “1” refers to a strong correlation, which means 2 labels of a given publication are semantically or syntactically linked to each other, such as relaying, concurrency, and coupling effects. Conversely, “0” indicates a weak association between multiple labels of a specific publication, and there is no obvious semantic or syntactic correlation.

## Discussion

### Principal Findings

There are several reasons for the “BERT + TextRNN” model to show optimal performance in cancer publication classification. First, cancer publications usually consist of long texts (eg, titles and abstracts) containing specialty terms and intensive contextual semantic correlations, which are quite suitable for the TextRNN model, which is good at processing sequential information with strong correlation and a high degree of uniformity. Moreover, comprehensive analysis of multilabel classification reflects that cancer publications are characterized by a high multilabel rate in areas with low research intensity due to the interdisciplinary and cooperative working, which enhances the contextual correlation to a certain extent. The “BERT + TextRNN” model is more likely to be efficient in such fields because it can effectively capture contextual semantics.

Compared with the TextRNN, the other models were insufficient and could be further improvement. The TextCNN might not be able to capture sufficient features, since it is not highly interpretable and well suited to address the fixed-length horizon issue. Although the DRNN is an enhanced version of the RNN with low computational speed, it fails to consider any upcoming input to the current state. Therefore, the DRNN is much less effective than the TextRNN. Being a long-linear model, FastText hardly handles the recognition of the long text of cancer publications and needs further optimization due to a limited recall rate.

### Limitations

The proposed classifier based on the “BERT + TextRNN” model has 2 issues. On the one hand, the performance of the classifier may be reduced due to the accumulation of errors caused by keyword extraction, which will be enhanced by adjusting the model parameters and adding a self-testing function. On the other hand, the tuple input of titles and abstracts was integrated to train the multilabel classifier, which proved to be better than the input of titles or abstracts alone. Therefore, cancer publications with both title and abstract are desired for the proposed classifier. However, for the few cancer publications papers without abstracts, the classifier we trained will still be usable and has a slight performance cost.

### Major Applications of the Proposed Classifier

We trained a classifier based on the “BERT + TextRNN” model for classifying the cancer literature at the publication level, which could directly assign multiple labels to each publication. The proposed classifier has at least 2 major applications. First, the desired model can achieve efficient and effective multilabel classification of cancer publications more granularly, not only for cancer publications in English, but also for full-text literature in other language whose titles and abstracts have English versions. Since the trained classifier is based on cancer publications with titles and abstracts, it should be suitable for any papers whose titles and abstracts are written in English (eg, Chinese medical publications). Another significant application is the fine-grained classification of scientific data on cancer research. Given that valuable data are accompanied by a brief description in English, the proposed model will classify them into the groups with appropriate CTs. Therefore, a content-based label in terms of CTs will be assigned to scientific data and literature, which provides a way to construct a full spectrum of data foundation for precision medicine.

### Conclusion

Given that existing classification methods are at the journal level and there is an urgent need for subject classification due to the proliferation of cancer research, a multilabel classifier was trained based on deep learning models, specifically “BERT + TextRNN.” Moreover, the proposed high-resolution classification model was evaluated as being efficient and effective for cancer publications in terms of quantitative comparison and feature analysis.

The innovative exploration in this study is as follows:

- The “BERT + TextRNN” classification model was trained for classifying cancer literature at the publication level, which shows promise in automatically assigning each publication at least 1 label to which it belongs.
- The proposed model achieves high-quality multilabel classification at the publication level, which could reflect the features of cancer publications more accurately with multiple labels compared to the existing method that annotates papers with a single label at the journal level.
- By comprehensive analysis of the correlation between multiple labels, as well as the data characteristics of multilabel cancer publications, the proposed model was verified to be suitable for the literature with features such as high specialization, uniform entity nouns, and standardized long texts.

In the future, the classification model will be extended to classify medical literature on cardiovascular disease and diabetes, where a great number of highly specialized publications have accumulated and are attracting increasing research attention, in order to improve health conditions worldwide.

## Abbreviations

BERT: Bidirectional Encoder Representation from Transformers
CNN: convolutional neural network
CT: cancer type
DPCNN: deep pyramid convolutional neural network
DRNN: deep recurrent neural network
ICD: International Classification of Diseases
ICD-10: International Classification of Diseases, Tenth Revision
ICRP CT: International Cancer Research Partnership Cancer Type
LSTM: long short-term memory
MLC: multilabel classification
MLTC: multilabel text classification
RNN: recurrent neural network
TextRNN: text recurrent neural network
TextCNN: text convolutional neural network
