# Correction: A Multilabel Text Classifier of Cancer Literature at the Publication Level: Methods Study of Medical Text Classification

**DOI:** 10.2196/62757

**Published:** 2024-06-05

**Authors:** Ying Zhang, Xiaoying Li, Yi Liu, Aihua Li, Xuemei Yang, Xiaoli Tang

**Affiliations:** 1 Institute of Medical Information Chinese Academy of Medical Sciences Beijing China

In “A Multilabel Text Classifier of Cancer Literature at the Publication Level: Methods Study of Medical Text Classification” (JMIR Med Inform 2023;11:e44892), the authors made one addition.

An Acknowledgments section was added to the paper, as follows:

This work was supported by the Innovation Fund for Medical Sciences of Chinese Academy of Medical Sciences (grant: 2021-I2M-1-033) and the Fundamental Research Funds for the Central Universities (grant:3332023163).

The correction will appear in the online version of the paper on the JMIR Publications website on June 5, 2024, together with the publication of this correction notice. Because this was made after submission to PubMed, PubMed Central, and other full-text repositories, the corrected article has also been resubmitted to those repositories.

